# Practical aspects of an on-line laboratory data system

**DOI:** 10.1155/S1463924679000742

**Published:** 1979

**Authors:** A. P. Rowland, C. C. Blake

**Affiliations:** 1 Analytical chemist Natural Environment Research Council Institute of Terrestrial Ecology, Merlewood Research Station Cumbria Grange-over-Sands LA11 6JU UK; 2 Research director Trivector Systems Ltd. York House, Stevenage Road Herts Hitchin UK


### Practical aspects of an on-line laboratory data system

A.P. Rowland

*Analytical chemist, Natural Environment Research Council, Institute of Terrestrial Ecology, Merlewood Research Station, Grange-over-Sands, Cumbria, LA11 6JU, U.K.*

C.C. Blake

*Research director, Trivector Systems Ltd., York House, Stevenage Road, Hitchin, Herts, U.K.*

### Introduction

A prime function of the Chemical Section of the Institute of Terrestrial Ecology is to provide a routine analytical service for ecologists. Their analytical requirements are varied, and include nutrient elements, structural components and pollutants in plant and animal materials, soils, rocks and natural waters. Because of the heterogeneity of biological systems, statistical considerations require the analysis of large numbers of samples. As many as ten thousand samples a year and up to ten constituents, generally inorganic nutrient elements, are determined on each sample.

The growing demand for an efficient analytical service made it necessary to look at alternative ways of improving throughput time. More efficient laboratory organisation, including the actual design of new accommodation, helped in this direction, but the most important factor was the use of automated analytical techniques.

The design and organisation of the laboratory allows the flexibility required for routine investigations, together with associated research projects. Most of the procedures in use are based on wet chemical methods, and the instrumental techniques most commonly in use are flame photometry, atomic absorption and continuous flow colorimetry, and these are fitted with fully automated sample presentation systems. The dual channel flame photometer is dedicated to the simultaneous determination of sodium and potassium. Atomic absorption is in very frequent use for calcium and magnesium and also for the minor nutrients of copper, zinc and manganese. A flameless atomiser attachment provides the facility to determine low levels of trace element and pollution constituents. Two automated colorimeter lines are reserved for nitrogen and phosphorus, whilst a third channel is available for other colorimetric methods e.g. nitrate, silicon and iron.

To keep abreast of the increased output from the instrumental techniques, clearly a corresponding improvement was needed for the data handling stage. This paper describes the development of a dedicated processing system for this purpose, one which has the merit of being used under heavy routine conditions for many months and has been proved to meet all demands placed upon it. A description of the assembly is included together with an assessment, allowing comparison with other on-line microprocessor systems and providing an insight into the structure for those thinking of acquiring equipment of this type.

### Specifications

Until recently a hybrid system based on the use of a chart recorder output, off-line to a computer, was in use in the laboratories at Merlewood Research Station [1]. In this system, the chart co-ordinates were fed into a computer using an x-y plotter and paper tape. Although this semi-automated system was not entirely ideal, experience in its use did provide a basis for the following specifications which would be required for a working data processing system:

(1) It should lead to a positive saving in staff time, allowing personnel to spend much more time on operations difficult to automate or else on non-routine tasks.

(2) It should be a desk top system suitable for installation in a chemical laboratory.

(3) It should be suitable for management by chemical staff with some experience in computer programming, although the routine operations would be done by non-technical staff.

(4) It should have facilities for data collection direct from instruments.

(5) It should have the capability of correcting for instrumental drift and provide adequate file handling facilities to allow calculation and manipulation of final results.

(6) It should incorporate a storage media suitable for analytical data and potentially suitable for some form of data bank.

It is well known that the advent of the microprocessor has resulted in desk top data processors with adequate storage facilities becoming available. Even though the microprocessor is now sufficiently low in cost to dedicate the control and processing of data from individual instruments, the need at Merlewood was for a system to process instrument signals directly from instruments and co-ordinate results from different sources. On-line data processing systems using small dedicated computers proved to be prohibitively expensive and somewhat inflexible. Several desk top computers are marketed which could be adapted for on-line data collection, but the necessary software is either unavailable or inflexible. In essence the requirements were for a micro-processor which would receive signals from several instruments simultaneously, including software, to collect, store and process data and additionally provide a high level language. A data processing package marketed by Trivector Systems Ltd. appeared to meet the main requirements and included proven software. The following account shows how this system was adapted to fulfil the specifications outlined above.

### Data processing system

The data processor is based on the Intel 8080 microprocessor chip and has 44K or store, a visual display unit (VDU), two dual floppy disk units, a high quality printer, a teletype (for balance control) and an eight channel multiplexed analogue to digital converter (ADC) (Figure 1). The disc (diskette) in drive 0 is used for storage of incoming data and that in drive 1 holds the previous day's data, although discs from earlier days may be loaded into drive 1 for processing, if required. Discs in drive 2 and 3 are reserved for application programs and resulting data.

The basic processor has the capacity to collect data from up to eight analogue instruments simultaneously. Special interfaces are provided to standardise the voltage output to the ADC. Each channel of the ADC, has a specific number providing a unique reference to that particular instrument.

Data are collected from each channel at the rate of one point every 250 millisecond, with eight points being averaged to give an effective rate of one smoothed point every two seconds. This rate of data collection is compatible with the rate of signal output for continuous flow colorimetry, and atomic absorption spectrophotometers, allowing complete definition of the fastest peaks to be obtained, without occupying an excessive amount of disc space. If instruments such as gas chromatographs were to be connected, a higher rate of data collection may have to be implemented.

Signals transmitted to the computer are also recorded simultaneously on a chart at the instrument, which enables the computer printout to be checked and provides a backup in case of computer failure.

An important feature of the Trivector package is the inclusion of the full facilities of the programming language BASIC, which may be used simultaneously with instrument data collection. This interactive language is easy to learn and has facilities for the solution of numerical problems, and these are utilised for general laboratory calculations, and further processing of collected data. Whilst discs 0 and 1 are solely for incoming data and system software, BASIC utilises discs 2 and 3. The system may also be used in the 'stand alone' mode for BASIC, to provide access to most of disc 0 and all of discs 1, 2 and 3.

Connected on-line to the data processor are two channels from a continuous flow colorimeter, and two from a dual element flame photometer. An automatic absorption spectrophotometer occupies a fifth channel. These were connected into the processor from the start, but modifications to the system have now extended the facilities to accent direct imput of sample weights onto the disc from two electronic balances. The system is designed so that the balances may be used simultaneously with any of the functions, such as collecting data, analysing data or running BASIC.

Incoming data to the system are stored in up to 120 of the 154 sectors on disc 0, leaving five sectors available for balance data, and the remainder for system software. The system software includes not only data collection facilities, but also programs for detecting peaks, estimating their height and correction for drift. The corrected file may be output to the printer or VDU before being stored on disc, and this file may then be manipulated using BASIC. Atomic absorption peaks are sorted and drift corrected using one program, whilst peaks obtained from automated colorimeters undergo a bubble spike rejection procedure before sorting and drift correction.

Setting up this 'turn key' system is almost as simple as switching on a calculator. At the beginning of the day, the disc in drive 0 contains the data collected the previous day and the disc in drive 1 the data collected two days previously. Both diskettes contain a copy of the program and system

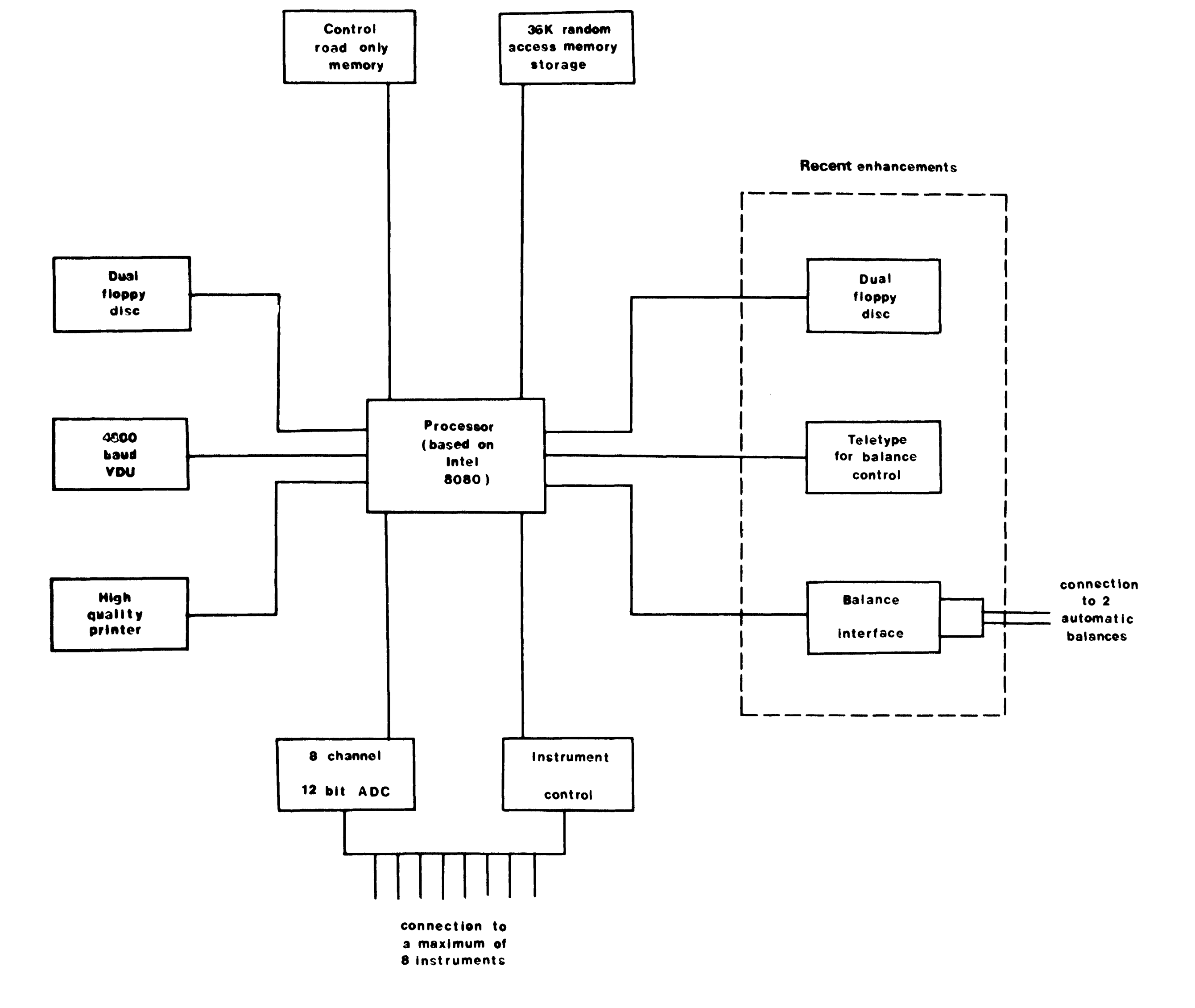

*Figure 1. Flow diagram of system hardware.*

constants. Upon switching on, all sectors of both discs are read and checked, with recoverable errors indicating the possibility of disc failure in the immediate future and irrecoverable errors signifying that disc corruptions have already occurred.

The disc in drive 1 may then be removed and stored, whilst another disc is inserted and the previous day's data copied on to it. This procedure clears the data from the disc in drive 0 to allow for collection of current data. If disc 0 is found to be unreliable during any of this checking, a new disc may be inserted and the program copied back from drive 1. By following this initial procedure each day, the chance of subsequent media failure and hence loss of data, is minimised.

Corruptions do occur occasionally, and it has been found to be essential to keep in reserve a clean master copy of the system software contained in disc 0. These discs are replaced at monthly intervals because of the limited life of floppy discs. Copy facilities are available on the system to provide new system masters and duplicate discs.

When the system is new, the 120 sectors on disc 0 may be arranged to cover suitable time periods from half an hour up to ten hours. Each sector gives a data collection time of approximately thirty minutes, and for each run an additional sector is also required to store the data following peak analysis of digitised data. Thus, a one hour run allocates three sectors (two for data collection and one for analysed data), and a two hour run occupies five sectors. Unless unusual batches are being analysed, it is seldom necessary to reconfigure the disc with regard to the time periods.

The master parameters of the system which have been defined carefully, include the start of data collection, the occurrence of a peak, the position of the first peak along with other factors concerned with sorting the digitised readings stored on the disc. Unlike the disc configuration parameter modification may be performed at any time, although they rarely need altering once they have been suitably defined. However, it sometimes occurs that an unusually 'poor' set of data cannot be analysed with the current parameters (e.g., because it is very noisy or the program indicates split peaks). The appropriate parameters may then be altered by the analyst to allow results to be obtained without having to re-run the samples on the instrument.

### Procedures for data collection from on-line instruments

When the laboratory instrument has been set up and linked to the processor the system is ready to accept details of the batch of samples to be analysed. Thirty-seven files are held on disc 0 and each may be structured according to the batch sequence with five codes relating to samples, standards and wash samples. Providing the channel is clear, the processor is available for initiation of data collection following input of sample cup detail number, cups per hour and appropriate identification codes. A specific record number is allocated to the batch of samples, provided storage space is available for that particular run length, and this run length is allocated according to the number of sample cups defined and the sampling speed, as cups per hour. The sampler and data link are commences simultaneously, but data may not arrive at the instrument detector for some time, e.g. from continuous flow analysers, so a time delay facility prevents undue waste of disc space before data arrives. An aborted run may be deleted from the disc at any time and a listing program indicates the status of the system when required. Subsequent reference to the batch of samples is by the record number and batches on disc 1 are accessed by adding one hundred to the record number.

Although there are facilities for up to 196 cups in any one run, samples are grouped into batches of not more than twenty five samples for reasons unrelated to the data handling needs. These, together with the appropriate standards and blanks make up a run. A typical sampling sequence for the determination of a constituent where only a small amount of drift is expected is included in Table 1. Experience has shown that calibration standards should be included more frequently when greater drift is expected. Calibration sample cups for instrumental gain and baseline drift are coded as 'C' and 'Z' peaks respectively, and the peak analysis utilises these controls to provide a drift corrected peak height output. Processing using the application programs compares the standard 'D' and sample 'S' peaks, whilst all wash 'W' peaks are ignored by application and system software. If during a run, erroneous peaks occur, the batch may be redefined with the particular cup to be excluded, being replaced by a wash cup. This is particularly important when errors occur in calibration samples.

**Table 1. A typical batch of samples**

| Sample Cup No. | Sample No. | Sample Code | |
|---|---|---|---|
| 1-2 | | C | Standard calibration |
| 3 | | W | Wash |
| 4-10 | | D | Standards |
| 11 | | W | Wash |
| 12-13 | | Z | Baseline calibration |
| 14-29 | 1-26 | S | Samples |
| 40 | | W | Wash |
| 41-43 | 27-29 | S | Samples (Control data) |
| 44-45 | | C | Standard calibration |
| 46 | | W | Wash |
| 47-48 | | Z | Baseline calibration |

### Peak analysis

On completion of the run, peaks are selected from the digitised data for subsequent peak height calculation using one of two system programs, either for flame or continuous flow instruments. The principles involved in the analysis of atomic spectroscopy peaks are similar in many respects to that of continuous flow peaks, except that no bubble spike testing or rejection is carried out, and also the use of an automatic sampler is optional. The analysis of atomic spectroscopy peaks is relatively simple, in that the program searches for a flat top peak with a low variation and the average of the flat portion is defined as the peak height.

The examination of continuous flow peaks is less straightforward as more factors have to be taken into account. These peaks are categorised as AA1 or AA2 style peaks, the main difference being that AA1 peaks are assumed 'pointed' and have a degree of symmetry about the maximum whereas AA2 peaks are assumed to rise slowly to an asymptotic value and then fall away more sharply (Figure 2). The output signal from continuous flow colorimetry is subject to interference due to air bubbles entering the flow cell, leading to transitory peaks being superimposed on to the normal signal, and an allowance for this has been made. The principles for peak analysis of either style of peak are similar and the analysis falls into six distinct stages:–

(1) Bubble spike rejection. This is done by making use of a seven point second order curve fit to predict subsequent points, in which any bubble spikes are completely sliced out. This is far superior to any filtering technique which will always have a residue of bubble spike signal.
(2) Calculation of initial baseline zero.
(3) Location of first peak. This must be a signal greater than a predetermined value, and may arrive at any time between one and eight minutes after the start of a run.
(4) Analysis of main peaks. This is carried out by evaluation of the portion of the peak currently under analysis with reference to the position of the previous error free peak, within the peak window. An error free AA1 peak is recognised only when:–

(a) there is a positive initial slope, a maximum and a negative final slope.
(b) there is a drop of a pre-determined value from the maximum for that peak.
(c) the slope does not again go positive, beyond the maximum.
(d) any air-bubble spikes which occur are less than 20 seconds wide.

The time at the maximum value, is then taken as the first approximation of the peak position, and a final estimate of height is calculated, predicting the position of the true maximum using the seven point quadratic centred on the first estimated maximum. Thus, the final value of the maximum, as evaluated, is more accurate than any of the individual data points read from the instrument.

The difference between AA1 and AA2 analysis is denoted by separate conditions for determining the peak. The main objective when processing AA2 style peaks, is to find the peak maximum where the change occurs from the slowly rising leading edge to the faster dropping trailing edge (knee). From the minimum following the peak trailing edge, the slope of the peak is recorded by searching backwards and the maximum is found either:–

(a) when the slope goes to zero, or
(b) when the slope becomes one eighth of the maximum, and no zero slope occurs within the next ten seconds of data.

Having found the knee, the peak height is then calculated by averaging a number of points in front of it. An abbreviated print out of an AA2 analysis is included in Table 2. Peak mode 1 indicates that the conditions for the peak analysis have been satisfied whilst peak mode 2 is listed when no peak is detected. Peak modes 3 to 6 are peak errors indicating unresolved peaks, split peaks, unresolved or incomplete peaks and extremely noisy peaks.

5. Calibration. Baseline drift is characterised by straight line segments joining the initial baseline and zero calibration values. Similarly, instrumental drift is assumed to be straight line segments joining the averaged values of group of standard calibration cups. The absolute value of these calibration cups need not be known exactly, but it must always be greater than any standard or sample in the batch. These calibrations are only used for drift correction. Each of the other types of cups are then calibrated, by a linear interpolation, as a percentage of the standard at that time, with the baseline deducted. Thus, complete allowance is made for both baseline and instrumental drift.
6. Print out of results. This is accompanied by a statistical summary and is available on the VDU or printer, with details of peak number, cup type, peak error code, peak height and calibrated peak height. Assuming no peak errors, the analysed data is stored on disc until standards and samples can be compared using BASIC. Alternatively this may be re-examined by alteration of either the run or the master parameters.

Table 2 illustrates an AA2 peak analysis from continuous flow colorimetry which has two types of errors present. Peak 26 has a peak error 3, indicating an indistinct knee. Standard peaks 4, 5 and 6 were noted as peak error 2 because of the low concentration range used in the chemical analysis. The output from the colorimeter was more typical of AA1 peaks, and analysis using the AA1 program, with a reduction of the master parameter controlling the level of peak detection, yields the data in table 3. The most common error, in general, with all forms of peak analysis is found when small spikes occur prior to the peak for atomic absorption analysis. Elimination of these spikes from the peak by reduction of the peak window, is required for a correct peak height analysis. Another problem which arose in the early days of the system occurred when samples with very low levels of ammonia-nitrogen in water were being analysed. Each of these peak heights was too low and registered peak error 2. The position of any following peak is calculated from a previous error free peak and the sampling speed (run parameter). Therefore the greater the number of consecutive peaks in error, the greater the accuracy of input of sampling speed required. As the timing devices on the automated sample presentation systems are insufficiently accurate for this purpose, each change in sampling rate is timed and, facilities have been added to the system for updating sampling rates. In general, it should be emphasised that re-examination of data was seldom necessary, since the establishment of the system.

In summary, the system software provides a drift corrected peak height output from continuous flow and atomic absorption or emission signals. Further manipulation of the data is then possible using the high level language facilities of BASIC.

**Balance operation**

Data from digital balances may also be collected simultaneously with the other functions such as collecting instrumental data, analysing data or running BASIC.

The system is designed to allow the weights from two electronic digital balances to be collected simultaneously on to disc for subsequent reading in BASIC, and includes a terminal located next to the balances for operator control. The weights are collected from the balances into the computer store in records, which may contain up to 60

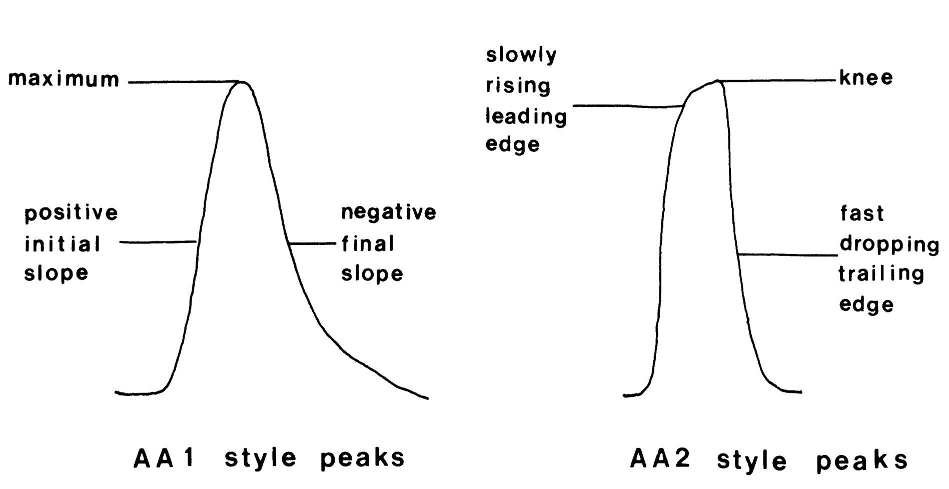

*Figure 2. Continuous flow peaks.*

**Table 2. Output from the AA2 peak analysis program**

| No. | Type | Mode | PK height | Calib PK Ht(%) |
|---|---|---|---|---|
| 1 | C | 1 | 124 | |
| 2 | C | 1 | 125 | |
| 3 | W | 2 | 64 | 1.25 |
| 4 | D | 2 | 64 | 1.06 |
| 5 | D | 2 | 68 | 6.39 |
| 6 | D | 2 | 74 | 13.89 |
| 7 | D | 1 | 38.5 | 31.48 |
| 8 | D | 1 | 103.5 | 48.30 |
| 9 | D | 1 | 118 | 63.26 |
| 10 | W | 1 | 147 | 93.40 |
| 11 | W | 2 | 66 | 2.31 |
| 12 | Z | 2 | 64 | |
| 13 | Z | 2 | 64 | |
| 14 | S | 1 | 86 | 21.27 |
| 15 | S | 1 | 86 | 20.43 |
| 16 | S | 1 | 101 | 33.51 |
| 17 | S | 1 | 111 | 42.36 |
| 18 | S | 1 | 109 | 38.29 |
| 19 | S | 1 | 217.5 | 129.43 |
| 20 | S | 1 | 217 | 125.48 |
| 21 | S | 1 | 207 | 114.06 |
| 22 | S | 1 | 214 | 116.60 |
| 23 | S | 1 | 215 | 114.40 |
| 24 | S | 1 | 219 | 114.56 |
| 25 | S | 1 | 210 | 105.17 |
| 26 | S | 3 | 236.5 | 121.67 |
| 27 | S | 1 | 237 | 119.20 |
| 28 | S | 1 | 251 | 126.11 |
| 29 | S | 1 | 209 | 95.09 |
| 30 | S | 1 | 195 | 83.77 |
| 31 | S | 1 | 200 | 85.17 |
| 32 | S | 1 | 184 | 73.22 |
| 33 | S | 1 | 206 | 85.32 |
| 34 | S | 1 | 159 | 54.96 |
| 35 | S | 1 | 181 | 66.95 |
| 36 | S | 1 | 179 | 64.41 |
| 37 | S | 1 | 217 | 85.02 |
| 38 | S | 1 | 206 | 77.18 |
| 39 | S | 1 | 95 | 14.01 |
| 40 | S | 1 | 200 | 71.02 |
| 41 | W | 2 | 72 | 0.95 |
| 42 | S | 2 | 72 | 0.82 |
| 43 | S | 2 | 72 | 0.69 |
| 44 | Z | 2 | 71 | |
| 45 | Z | 2 | 71 | |
| 46 | C | 1 | 274 | |
| 47 | C | 1 | 275.5 | |

**Table 3. Re-Analysis of table 2 data using AA1 peak analysis**

| No. | Type | Mode | PK height | Calib PK Ht(%) |
|---|---|---|---|---|
| 1 | C | 1 | 125.5 | |
| 2 | C | 1 | 126.5 | |
| 3 | W | 2 | 65 | 2.44 |
| 4 | D | 2 | 64.5 | 1.38 |
| 5 | D | 1 | 69.5 | 7.84 |
| 6 | D | 1 | 75.5 | 15.06 |
| 7 | D | 1 | 90.5 | 32.90 |
| 8 | D | 1 | 104.5 | 48.18 |
| 9 | D | 1 | 119.5 | 63.49 |
| 10 | W | 1 | 148 | 92.73 |
| 11 | W | 2 | 67 | 2.43 |
| 12 | Z | 2 | 65 | |
| 13 | Z | 2 | 65 | |
| 14 | S | 1 | 87.5 | 21.60 |
| 15 | S | 1 | 87.5 | 20.75 |
| 16 | S | 1 | 102.5 | 33.72 |
| 17 | S | 1 | 112.5 | 41.51 |
| 18 | S | 1 | 110.5 | 38.45 |
| 19 | S | 1 | 219.5 | 129.37 |
| 20 | S | 1 | 220.5 | 126.66 |
| 21 | S | 1 | 209.5 | 114.45 |
| 22 | S | 1 | 216.5 | 116.96 |
| 23 | S | 1 | 217.5 | 114.75 |
| 24 | S | 1 | 220.5 | 114.13 |
| 25 | S | 1 | 211.5 | 104.80 |
| 26 | S | 1 | 238.5 | 121.53 |
| 27 | S | 1 | 238.5 | 118.73 |
| 28 | S | 1 | 254 | 126.60 |
| 29 | S | 1 | 210.5 | 94.77 |
| 30 | S | 1 | 196.25 | 83.35 |
| 31 | S | 1 | 201.5 | 84.90 |
| 32 | S | 1 | 184.5 | 72.41 |
| 33 | S | 1 | 207.5 | 85.03 |
| 34 | S | 1 | 160.5 | 54.88 |
| 35 | S | 1 | 182.5 | 66.79 |
| 36 | S | 1 | 180.5 | 64.27 |
| 37 | S | 1 | 218.5 | 84.73 |
| 38 | S | 1 | 208.5 | 77.48 |
| 39 | S | 1 | 96.5 | 14.19 |
| 40 | S | 1 | 201.5 | 70.81 |
| 41 | W | 1 | 72 | 0.41 |
| 42 | S | 2 | 73.5 | 1.07 |
| 43 | S | 1 | 74.5 | 1.46 |
| 44 | Z | 2 | 72 | |
| 45 | Z | 2 | 72 | |
| 46 | C | 1 | 277 | |
| 47 | C | 1 | 277.25 | |

weights. A record is initiated for a batch of weights by assigning the weights to a balance which has not been allocated.

Associated with each balance is a small box containing a lamp and a button. When the computer is ready to take a reading from the balance, the lamp will be illuminated, the reading itself being initiated by pressing the button. The reading is subsequently printed out on the terminal and stored in the computer store until completion of the weight record, when the record is written to the disc in drive 0. Further weighings may be added to an existing record on disc 0, or individual weighings may be altered by manual input. In addition, extra weights may be added to an existing record.

Once a record has been stored on disc 0, it is retained until deleted by the operator. When disc 0 is full and instrumental data is transferred to disc 1, the balance data remains on disc 0 until each record is deleted. This allows batches of weighings from previous days to be completed without inconvenience, and allows efficient processing of data without error prone file manipulation.

Each record stored on disc 0 also contains background information relating to that batch of weighings. Several of these items identify the file, while others are relevant for subsequent data processing using BASIC. System software is provided for listing all record headings for system management or listing an individual record for information.

### BASIC

The system includes a BASIC interpreter which may be entered at any time whilst the system is running, with 16K of store available for user programs at present. Interruption of programs for initiation of data collection or analysis of raw data does not result in loss of BASIC data, as on subsequent re-entry, the program is continued exactly as it was left.

A powerful feature of BASIC is its suitability for accessing floppy discs for both data and program storage. Data storage is accomplished by reading or writing arrays on to disc, whereas the program storage facility writes the program to disc and reads from disc entering at line 0. This enables full overlaying of programs as well as the ability to read programs directly from the disc at any time. For simplification, the application programs in BASIC are stored on disc 2 whilst the data processed using these programs is stored on the disc in drive 3. This provides adequate storage space for all applications programs in routine use and sufficient data storage for at least one month's data.

Included in BASIC is a command to give access to the results of peak height analysis performed by the system software, giving access to the information, for example in Table 2. This enables further manipulation of the drift corrected peak height data into a suitable analytical form.

Filing in BASIC is by location, necessitating storage of background information associated with the data in order to keep a file of all processed data on that disc. This is essential for ease of manipulation.

Several linear and curvilinear regression programs have been designed to process either drift corrected data recalled from disc, or manual input data obtained from the instruments at present not connected to the processor. In the curvilinear regression program for on-line data, the record number of the drift corrected peak height data file initiates the reading of the file. The value of the standards may then be input, and the standards are sorted ("D" peaks) and a curvilinear regression is performed. Following output of the regression constants, the standard peak heights are predicted using the regression constants. Standards in error may be eliminated from the regression, and when the standard predictions are satisfactory, all sample peaks ("S" peaks) are evaluated using the regression constants. Included with each batch of samples is a reference sample and a duplicate, whose values are checked manually to ensure that the precision falls within acceptable limits. The data from this program and its background information are written to disc for storage so that the next program may be chained using the OVER statement. This clears the existing program and variables from the store before calling another program from the disc and commencing at line 0. Figure 3 illustrates the sequence of programs at present used to process the drift corrected data, and table 4 gives an example of the BASIC output from total phosphorus estimation on vegetation.

BASIC also contains a command gaining access to the balance records for further processing. Commonly the balance data are used directly from weight records in calculations, to give results in %, mg $100g^{-1}$ or $\mu g\ g^{-1}$. However, in the event of heavy demand on the balances, the balance data is written on to disc in BASIC releasing the record for further weighings. The BASIC weight file may then be used for calculations.

Weighings are assumed to fall into two distinct categories – mode 1 and mode 2. Mode 1 weight records are constituted

**Table 4. Processed data from BASIC**

Record No. 119

| Standard values | Standard predictions (from mathematical equation) |
|---|---|
| 0 | 0.036 |
| 0.5 | 0.477 |
| 1 | 0.962 |
| 2 | 1.993 |
| 3 | 3.035 |
| 4 | 4.037 |
| 5 | 4.961 |

Equation used: 0.0 + 0.315X + 0.022XX

Wt. Vol. & Dilution constant for batch

Wt. = 0.4g   Vol. = 50ml   Dil. = *25

M1245   Total-P   3/5/79

| | PPM | PPM-Blanks | % |
|---|---|---|---|
| 1 | 0.7941 | 0.756 | 0.236 |
| 2 | 0.7471 | 0.7089 | 0.222 |
| 3 | 0.743 | 0.7048 | 0.22 |
| 4 | 0.8906 | 0.8528 | 0.266 |
| 5 | 0.6821 | 0.6439 | 0.201 |
| 6 | 0.699 | 0.6608 | 0.207 |
| 7 | 0.9746 | 0.9364 | 0.293 |
| 8 | 0.7471 | 0.7089 | 0.222 |
| 9 | 1.2093 | 1.2712 | 0.397 |
| 10 | 1.126 | 1.0879 | 0.34 |
| 11 | 1.212 | 1.1738 | 0.367 |
| 12 | 1.0067 | 0.9685 | 0.303 |
| 13 | 1.356 | 1.3178 | 0.412 |
| 14 | 1.56 | 1.5218 | 0.476 |
| 15 | 1.0498 | 1.0117 | 0.316 |
| 16 | 0.9098 | 0.8716 | 0.272 |
| 17 | 0.7209 | 0.6827 | 0.213 |
| 18 | 0.8206 | 0.7824 | 0.245 |
| 19 | 1.5234 | 1.4852 | 0.464 |
| 20 | 1.4016 | 1.3634 | 0.426 |
| 21 | 1.3394 | 1.3012 | 0.407 |
| 22 | 0.6135 | 0.5754 | 0.18 |
| 23 | 1.0493 | 1.0111 | 0.316 |
| 24 | 1.0067 | 0.9685 | 0.303 |
| 25 | 1.3783 | 1.3401 | 0.419 |

**Table 5. Print out form a data bank search**

All results are expressed on a dry Wt. basis & are normally %
except for Cu, Zn & other trace elements which are µg/g

*Erica Cinerea* (current years growth)

| Site name | Grid Ref. | Date | Na | K | Ca | Mg | Fe | Mn | Cu | Zn | P | N |
|---|---|---|---|---|---|---|---|---|---|---|---|---|
| Hartland | SY9585 | 00/07/69 | 0.102 | 0.45 | 0.25 | 0.161 | 0.005 | 0.003 | 4.91 | 12.9 | 0.043 | 0.63 |
| Westleton | TM4680 | 00/07/69 | 0.045 | 0.53 | 0.22 | 0.129 | 0.004 | 0.018 | 3.24 | 14.7 | 0.083 | 0.6 |
| Porthtowan | SW7047 | 00/07/69 | 0.22 | 0.59 | 0.23 | 0.118 | 0.008 | 0.018 | 7.12 | 15.1 | 0.037 | 0.76 |
| St. Kilda | NF1099 | 00/07/69 | 0.156 | 0.29 | 0.29 | 0.193 | 0.005 | 0.005 | 3.4 | 15.5 | 0.04 | 0.69 |
| Glascwm | SO1753 | 00/07/71 | 0.01 | 0.77 | 0.55 | 0.185 | 0.005 | 0.056 | 5.58 | 15 | 0.121 | 1.08 |
| Faichem | NH2902 | 00/07/71 | 0.148 | 0.6 | 0.4 | 0.22 | 0.005 | 0.015 | 5.5 | 9.17 | 0.064 | 1.06 |
| Glengouland | NN7653 | 00/07/71 | 0.092 | 0.56 | 0.38 | 0.21 | 0.005 | 0.015 | 5.5 | 21.3 | 0.056 | 0.85 |
| Lund | SE6190 | 00/07/71 | 0.083 | 0.88 | 0.34 | 0.159 | 0.031 | 0.026 | 11.3 | 38.8 | 0.092 | 1.05 |
| Holt Heath | SU0604 | 00/07/70 | 0.073 | 0.7 | 0.38 | 0.178 | 0.011 | 0.009 | 7.63 | 28.8 | 0.041 | 0.95 |
| Brownhills | SK0406 | 00/07/70 | 0.061 | 0.79 | 0.45 | 0.137 | 0.02 | 0.022 | 9.71 | 102 | 0.097 | 0.8 |
| | Mean | | 0.099 | 0.616 | 0.349 | 0.169 | 0.01 | 0.019 | 6.39 | 27.3 | 0.067 | 0.793 |
| | Std. Dev. | | 0.013 | 0.058 | 0.034 | 0.011 | 0.003 | 0.001 | 1.11 | 24.9 | 0.01 | 0.002 |

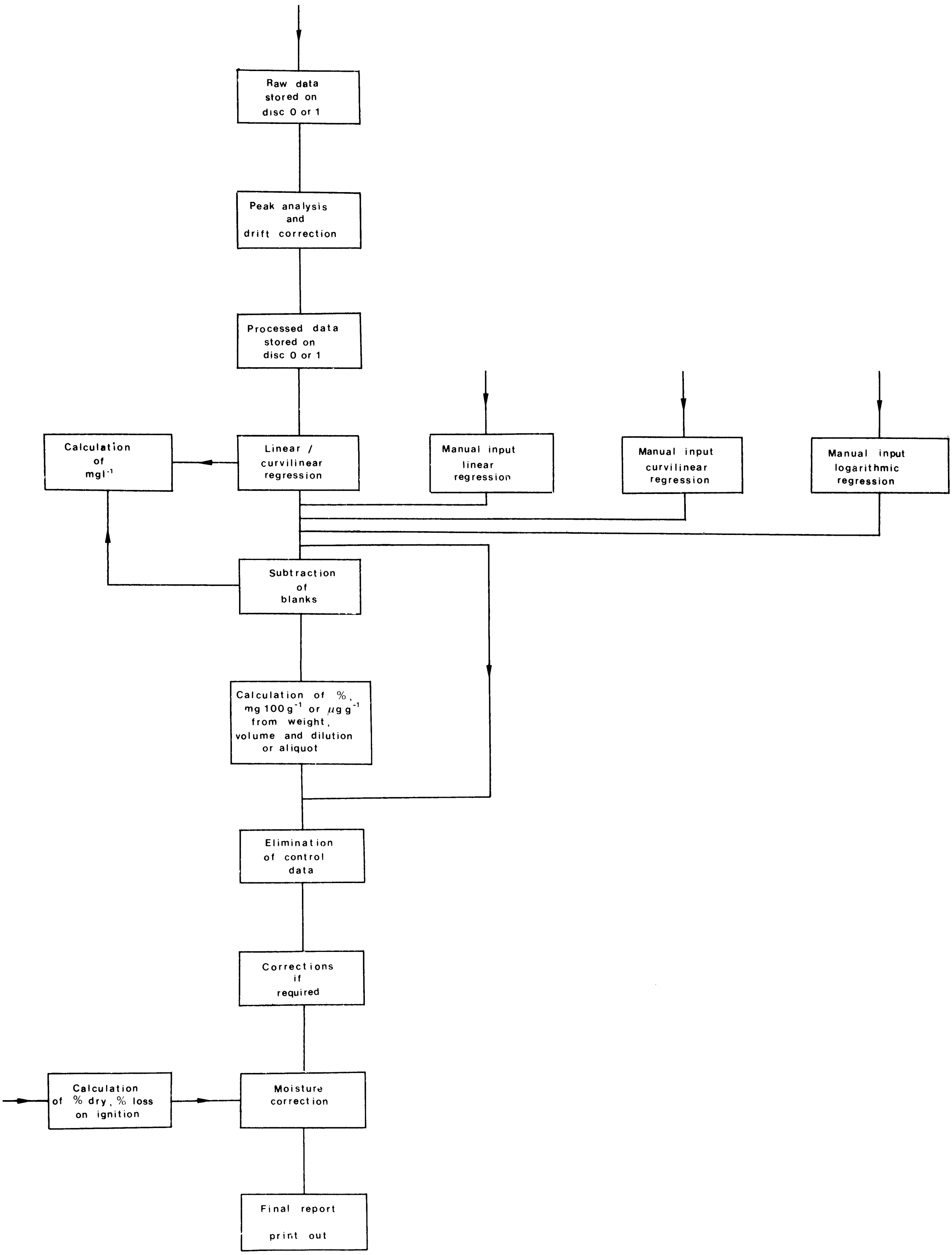

*Figure 3. Flow diagram of system software.*

with one weight per sample, e.g. weight of sample (scoop tared). Mode 2 records contain two weights per sample, e.g. (a) weight of crucible and (b) weight of crucible + sample. The file number is used to identify the order in which a particular determination has proceeded, e.g. in moisture determination, file 1 contains a batch of mode 2 weights (weight of crucible and weight of crucible + sample) and file 2 contains a batch of mode 1 weights (weight crucible + oven dried sample). Suitable applications programs have been designed to sort and process the data and store the weights and results in BASIC, thus releasing the weight record.

On conclusion of calculations relating to a batch of samples, all the results are already stored on disc 3 together with relevant background information. A versatile program for report compilation searches for completed calculations, and this data is then presented in tabulated form. At this stage data may be modified, e.g. 'less than' figures inserted, and also data may be added directly for determinations which have not required computation, e.g. pH determinations.

The laboratory data processing system has 16K of core and 308K on each of the two discs for BASIC, enabling some data storage and statistical analysis of data encountered in the analysis of biological materials, to be performed simultaneously with instrument data collection. However, a 'stand alone' facility has been provided to give the user 26K of store and 300K of data on discs 1, 2 and 3, and 250K on disc 0.This provides adequate processing facilities for the storage and retrieval of selected chemical data accumulated over many years. Because of the limitations in speed on disc search, the input of data to this data bank is in a very selective form to reduce searching time. A tabulated print out of data from a data bank search on *Erica conerea* is contained in Table 5.

### Discussion

The data processing system which has been installed meets all of the specifications previously outlined. In practice a few functions of the system do not fit in with existing laboratory practices, and these were modified accordingly as the problems manifested themselves. For example, the timing mechanisms on the automated samplers were not sufficiently precise, and the software was modified to allow for minor deviations in sample cup handling rate.

The saving in man-hours is of considerable importance when assessing the impact of a microprocessor system. In the calculation stages, the processing of data has now become less labour intensive, although the principal saving has been in the time spent on checking procedures. This has been brought about because these are no longer a matter of re-calculation, but of comparing calculated results with the chart output from the instruments. Initial experience with the system has highlighted the importance of this particular check.

With the advent of electronic digital balances, the addition of two digital balances to this system has been a simple method for improvement of laboratory automation. Obviously, this has removed certain operator errors normally associated with weighing and increased the efficiency of data processing. The time spent in the process of weighing samples has also been reduced. These advantages combined have proved a worthwhile addition to the system.

A major impact of the use of the microprocessor based system has been on laboratory operations, not least through the need to re-arrange the sample processing sequences. Some flexibility has been lost due to the requirement of having to inform the processor of the number of sample cups being fed into the instrument, and their sequence on the sampler turntable. Once the run length has been allocated, this may not be altered. Also, the analysis of a further batch may not commence until data collection from the previous batch has

**Table 6. Silicon analysis data (mg $l^{-1}$)**

| Standard Concentration | Peak Height (arbitrary units) | Standard predictions using 0-6 Stds | 0-3 Stds | 3-6 Stds |
|---|---|---|---|---|
| 0.00 | 6.25 | 0.12 | 0.01 | -0.41 |
| 1.00 | 160.1 | 0.82 | 0.98 | 0.37 |
| 2.00 | 262.8 | 1.88 | 2.02 | 1.54 |
| 3.00 | 360.5 | 3.20 | 2.99 | 3.00 |
| 4.00 | 416.8 | 4.09 | 3.56 | 3.99 |
| 5.00 | 468.6 | 5.00 | 4.08 | 5.01 |
| 6.00 | 515.3 | 5.89 | 4.55 | 6.00 |

been completed. However, it was possible to remove these problems by reorganising the laboratory routine. Potentially, more serious, is the direct impact of the microprocessor for the personnel involved. Past experience indicated that involvement in the calculating stages provided a welcome contrast from other laboratory duties for the assistant staff. Clearly, this aspect needs careful consideration and any implications on staff morale must be taken into account before the introduction of a fully on-line data processing and handling system. Participation of staff at all levels in the commissioning of the processor and design of the BASIC programs has contributed towards the successful introduction of this system in the authors' laboratory.

After the initial commissioning stages had been completed, the system has been managed and operated by staff who had no previous computing experience. For a period of two months after the introduction of the new system, all calculations were checked using the previous semi-automated procedures. These comparisons indicated that improved accuracy has been achieved with the introduction of the microprocessor, mainly by removal of systematic errors. Any discrepancies could generally be ascribed to faults arising in the laboratory chemical procedures.

For example, the molybdenum blue colorimetric procedure for silicon, had linear output up to 3 ppm silicon, with slight curvilinearity above this value (Table 6). This situation produced a non-mathematical curve using all the standards in the regression. This is illustrated in column 3 of Table 6 by the unsatisfactory prediction of the standards over the whole range. Previously, manual graphical methods automatically compensated for minor tendencies towards curvilinearity. Problems of this nature must be corrected by modification of the procedure to ensure application of the correct chemistry using the appropriate range of standards, sample concentration and other instrumental conditions. Such data was processed by elimination of the first or last three standards from the regression and applying this regression to the appropriate samples. Column 3 illustrates the prediction of standard values using all the standards, and columns 4 and 5 show the effect of the regressions on the standard values from 0-3 ppm Si and 3-6 ppm Si respectively.

In addition to the savings in manpower from the increased speed and efficiency of the new data processing system, it is also advantageous to have quick access to the final chemical data. Storage facilities on this system are provided by floppy diskettes. This storage media is different from magnetic tape or paper tape, the life of each disc being limited, especially if one portion is frequently used. Experience showed it was a false economy to use inferior discs. Good quality discs should be used and these must be handled with care both in loading and storage.

For most operations involving wet chemistry, initial treatments including weighing, making up to volume, reagent addition and dilution have to be included in the calculation of the final result. These variables can be entered into the central processor. In addition, the assembly of all sample results and the co-ordination of calculations for a final

Table 7. Customer report

| Chemical data | | | |
|---|---|---|---|
| Vegetation | M1275 | % | 13. 7.79 |
| | Na | K | Ca |
| 1 | 0.017 | 1.4 | 1.4 |
| 2 | 0.011 | 1.3 | 1.5 |
| 3 | 0.015 | 1.4 | 1.4 |
| 4 | 0.055 | 2.7 | 0.68 |
| 5 | 0.073 | 1.8 | 1.2 |
| 6 | 0.064 | 2.0 | 0.79 |
| 7 | 0.011 | 1.1 | 0.31 |
| 8 | 0.021 | 2.6 | 0.69 |
| 9 | 0.026 | 2.3 | 0.71 |
| 10 | 0.45 | 2.4 | 1.6 |
| 11 | 0.45 | 2.6 | 1.5 |
| 12 | 0.45 | 2.7 | 1.2 |
| 13 | 0.015 | 2.0 | 0.61 |
| 14 | 0.021 | 2.1 | 0.59 |
| 15 | 0.009 | 2.2 | 0.84 |
| 16 | 0.10 | 3.6 | 0.56 |
| 17 | 0.10 | 3.0 | 0.51 |
| 18 | 0.10 | 3.2 | 0.38 |
| 19 | 0.013 | 3.1 | 2.1 |
| 20 | 0.017 | 2.6 | 0.53 |
| 21 | 0.009 | 2.8 | 0.65 |
| 22 | 0.008 | 2.7 | 0.68 |
| 23 | 0.035 | 2.1 | 0.58 |
| 24 | 0.061 | 2.0 | 0.42 |
| 25 | 0.029 | 2.2 | 0.43 |

Institute of Terrestrial Ecology
Subdivision of Chemistry and Instrumentation
Merlewood Research Station
Grange-Over-Sands
Cumbria
LA11 6JU

customer report can be produced directly by the system, an example of which is included in Table 7.

Many instruments are now marketed incorporating a dedicated microprocessor. These can be linked into the system described by processing the analogue signals using the instrument microprocessor prior to transfer of the digital readings to the central processing system for further processing, report compilation and archiving.

Despite the relatively small capacity of this data processing system, it has been possible to design a data bank for use in the "stand alone" mode, utilising all of disc 1 for record storage. Input of data via the VDU is quick and efficient and several search programs have been designed to extract data from the discs in the appropriate form. In order to contain as many records as possible on each disc, data has been input as codewords onto disc. Micro-computers will generally run slower than large main-frame computers, as pointed out by Little and Reeves (2), and this should be borne in mind when planning an ambitious data bank utilising a small system. Input of data has to be rigidly standardised in order to minimise this problem.

REFERENCES
[1] Allen, S.E., Grimshaw, H.M., Parkinson, J.A., Quarmby, C., "Chemical Analysis of Ecological Materials", Blackwell.
[2] Littler, J.S. and Reeves, R.M., **Chemistry in Britain.** 1978, 3, 118-126.

### An automatic monitor for lead emissions from stacks: design philosophy and preliminary evaluation*

C.J. Jackson

*Health & Safety Executive, 403 Edgware Road, London NW2 6LN, UK.*

### Introduction

In 1974, the Alkali Inspectorate, at that time part of the Department of the Environment (DOE), concluded that there was a need for a device to continuously monitor metal dust and fume emissions from stacks and ducts. As a first requirement it was decided to investigate the continuous monitoring of emissions from the lead smelting process. Lead and its compounds are major industrial and environmental hazards and emissions of lead fume and dust are monitored against standards set in a Health and Safety Executive Code of Practice for Lead Works.

** This paper was originally presented at a Chemical Society, Analytical Division, Automatic Methods Group meeting entitled 'Safety and Automation' at Chester, 5 October 1978.*

A contract was, therefore, concluded between DOE and BNF Metals Technology Centre (BNF), for the latter organisation to develop a continuous monitor for lead emissions based on the requirements given in the, at that time, proposed Code of Practice. Indeed, the specific instrument performance was defined as:

(i) To record, reasonably accurately, the level of emission which occurs during normal operation.
(ii) To detect any significant increases in the emissions due to failure of the filter bags in the fume arrestment plant.
(iii) To give a positive meaningful reading within the normal concentration limit of 0.02 g $M^{-3}$.
iv To give a rapid response to concentration up to 0.10g $M^{-3}$.

Finally it was required that such an instrument be designed

